# University of Buffalo 1846—1910

**Published:** 1910-07

**Authors:** 


### University of Buffalo 1846—1910

THE sixty-fourth annual commencement of the department of medicine, the twenty-third annual commencement of the department of pharmacy, the twenty-second annual commencement of the department of law and the eighteenth annual commencement of the department of dentistry of the University of Buffalo, took place at the Teck Theater, Friday, May 27, 1910, at 11 A. M. The preliminary announcement of the exercises during commencement week was published in the June issue of the JOURNAL. The magazine, however, went to press too early to give the full proceedings, hence we publish them in this edition, thus completing the historic sequence.

MEDICAL ALUMNI ASSOCIATION.

This association, organized in 1874, held its thirty-fifth annual meeting, during commencement week. The program for Tuesday, May 24, was as follows:

10.00 A. M.—Clinical lectures and demonstrations by various specialists, at the Buffalo General Hospital.

4.00 P. M.—First lecture of the Harrington series, by Dr. J. Clarence Webster, Professor of Obstetrics and Gynecology, Rush Medical College, Chicago. Subject of the Harrington Lectures—"Some Phases of Abdominal and Pelvic Diseases in Women," at alumni hall, college building.

8.00 P. M.—The classes of '50, '60, '70, '80, '90 and 1900 met in the college building for organization and registration.

8.30 P. M.—Business meeting and election of officers.

9.00 P. M.—President Henry J. Nichols, '82, delivered the annual address.

Dr. William H. Bergtold, '86, of Denver, read a paper on "Family Infection—706 Cases of Tuberculosis."

10.30 P. M.—A supper and smoker, were given in the college library.

WEDNESDAY, MAY 25.

9.00 A. M., 1.00 P. M.—Medical clinics, at the Buffalo General Hospital. Cases to be presented and the names of the clinicians posted on the bulletin board in the College Building.

3.00 P. M.—Second Lecture of the Harrington series by Dr. J. Clarence Webster, at alumni hall.

4.00 P. M.—New Surgical Technic for Operations on the Liver, by Jacob Frank, '82, of Chicago, at alumni hall.

The evening was devoted to the entertainment by the various fraternities of their alumni and guests of the association.

THURSDAY, MAY 26.

9.00 A. M., 1.00 P. M.—Surgical clinics, at the Buffalo General Hospital.

3.00 P. M.—Third lecture of the Harrington series by Dr. J. Clarence Webster, at alumni hall.

4.00 P. M.—Stomach Disorders Requiring Surgical Intervention from the Viewpoint of an Internist, by Charles D. Aaron, '88, Detroit, at alumni hall.

7.00 P. M.—Complimentary dinner to Dr. Matthew D. Mann, retiring Dean of the Medical Department and Professor of Obstetrics and Gynecology, was given at the Lafayette Hotel.

FRIDAY, MAY 27.

11.00 A. M.—Sixty-fourth annual commencement exercises of the University of Buffalo, including the departments of medicine, law, pharmacy and dentistry, were held at the Teck Theater, Main and Edward Streets.

Matthew D. Mann, A. M., M. D., delivered the address.

The following order of exercises was observed:

Music.

Song—*"Alma Mater."*

Where once the Indian trod the silent wood,
Above the beach where antlered deer have stood,
Where martyrs brought the faith, and patriot swords
Assembled oft to repel invading hordes.

CHORUS:

Brothers, tonight we sing the chorus free,
Pledging the health of our University,
To U. of B., to U. of B.,
Our Alma Mater by the inland sea.

Before the Saxon march the forest fell;
The Church, the School, the Shop their story tell;
Off wind-swept beach proud ships securely ride,
Here Peace hath blest and Plenty shall abide.
[CHORUS

Beside Lake Erie, where the daring deep,
The con'nent's erring child, hastes to the leap,
And crushing cliffs in youthful, eager quest,
From rock to rock leaps to her ocean rest.
[CHORUS

Prayer by Rev. J. A. Regester, D.D.

Presentation to the Chancellor, Charles P. Norton, A.B., of candidates for the degree of Doctor of Medicine.

Administration to the class of the Hippocratic oath.

Conferring the degree.

Announcement of honors.

Presentation of candidates for the degree of Bachelor of Pharmacy.

Conferring the degree.

Presentation of candidates for the degree of Doctor of Pharmacy.

Conferring the degree.

Presentation of candidates for the degree of Analytical Chemist.

Conferring the degree.

Announcement of honors and prizes.

Presentation of candidates for the degree of Bachelor of Laws

Conferring the degree.

Announcement of honors and prizes.

Presentation of candidates for the degree of doctor of Dental Surgery.

Conferring the degree.

Announcement of honors.

Music.

Address by Matthew D. Mann, A.M., M.D.

Benediction.

Music.

### CONFERRING THE DEGREE.

The degree of Doctor of Medicine was conferred by Chancellor Charles P. Norton upon:

| | |
|---|---|
| Baeslack, Frederick Wm., B.A., M.A. | 240 Landon St., Buffalo. |
| Blum, Raymond Joseph | 273 Main St., Dansville, N. Y. |
| Brown, Stanley John | Nunda, N. Y. |
| Burke, Thomas Joseph | Belfast, N. Y. |
| Carey, Clyde Lorenzo | 321 Madison Ave., Elmira, N. Y. |
| Culkowski, Anthony Stanislaus | 94 Swinburne St., Buffalo. |
| Duffy, Benedict James | 510 Wayne St., Olean, N. Y. |
| Eustace, Charles William | 101 Seymour St., Buffalo. |
| Fakhoury, Elias Khalil | Nazlet-Sow (Bierut), Egypt. |
| Goodell, Charles Ellsworth | Springville, N. Y. |
| Greene, Clayton Wellington, A.B. | 385 Jersey St., Buffalo. |
| Grenolds, James Walter | Rexville, N. Y. |
| Harris, Jennie Harper | 96 Seymour St., Tonawanda. |
| Hartigan, William Stephen | 409 Powell St., Elmira, N. Y. |
| Hoehn, Frank Victor, A.B. | 931 Main St., Buffalo. |
| Hogan, John Vincent | 1807 Sixteenth St., Niagara Falls. |
| Hughes, Ralph Raymond | 707 Crescent Ave., Buffalo. |
| Juhre, Roy John | 768 Greenwood Ave., Buffalo. |
| Kavinoky, Nadina Reinstein | 576 Jefferson St., Buffalo. |
| Keyes, Marion Alvah, Jr., L.L.B. | Mayville, N. Y. |
| Klein, Julius James, M.D. | 24 Woodlawn Ave., Buffalo. |
| Kurtz, Nellie Ettie | 432 Porter Ave., Buffalo. |
| McColl, James MacPherson | 7 Tremont St., N. Tonawanda. |
| McMahon, Michael James | Mt. Jewett, Pa. |
| Nesbitt, Clarence Clark | Albion, N. Y. |
| Orvis, Howard Andrew | Orchard Park, N. Y. |
| Paxton, Roy A. | 83 Putnam St., Buffalo. |
| Rebescher, Otto I. | 182 Rose St., Buffalo. |
| Saylin, George J. | 198 Spring St., Buffalo. |
| Sperry, Frederick Edward | 105 Florida St., Buffalo. |
| Stein, August Howard | 122 Dodge St., Buffalo. |
| Stockwell, Raymond Wm. | R.F.D. No. 11, Lockport, N. Y. |
| Strozzi, Frederick Ettore | 53 Front Ave., Buffalo. |
| Stygall, James Henry | 44 Normal Ave., Buffalo. |
| Sullivan, James Cornelius | 5 River St., Olean, N. Y. |
| Vanderboget, Carlton Lakey | Palmyra, N. Y. |
| Wagner, Albert Warren | 367 High St., Buffalo. |
| Wilkins, Leslie Mayo | Nunda, N. Y. |

Chancellor Norton also conferred the degree of bachelor of pharmacy upon forty candidates; the degree of doctor of pharmacy upon Charles W. Bullock; the degree of analytical chemist on nine candidates; the degree of bachelor of laws upon thirty-seven candidates; and the degree of doctor of dental surgery upon seventeen candidates. These added to the thirty-eight medical graduates make a total of 142 degrees conferred at the commencement of 1910.

### NOTES OF THE WEEK

The medical department does not grant prizes but instead confers distinction upon the six standing highest in the class for all reasons. The "honor men," as they are termed, for 1910, were James Cornelius Sullivan, George J. Saylin, Benedict James

Duffy, Clayton Wellington Greene, A.B., Frederick Ettore Strozzi, Charles Ellsworth Goodell.

The most important event of the alumni meeting was the Harrington lectures delivered by Dr. J. Clarence Webster, of Chicago. The distinguished lecturer not only interested and instructed his audiences, but through his geniality of manner and courteous bearing made many warm friends.

### Alumni Officers 1910-11.

At the alumni election held Tuesday evening, the following named were chosen:

President, Marshall Clinton, '95, Buffalo; first vice-president, Charles L. Preisch, '98, Lockport; second vice-president, Nellie V. Chappell, '92, Buffalo; third vice-president, Eugene B. Horton, '02, Niagara Falls; fourth vice-president, Edgar A. Forsyth, '89, Buffalo; fifth vice-president, Jacob E. K. Morris, '79, Olean, N. Y.; treasurer, Herman K. DeGroat, '97, Buffalo; secretary, Franklin W. Barrows, '93, Buffalo.

*Trustees.*—Robert P. Bush, '74, Horseheads, N. Y.; Fridolin Thoma, '87, Buffalo; DeLancey Rochester, '84, Buffalo; Albert T. Lytle, '93, Buffalo; Henry J. Nichols, '82, Bradford, Pa.

*Executive Committee.*—Frederick J. Parmenter, '03, chairman, Buffalo; Lawrence Hendee, '97, Buffalo; Lesser Kauffman, '04, Buffalo; Frederick C. Busch, '97, *ex-officio,* Buffalo; Eli H. Long, *ex-officio,* Buffalo.

The class of '70 held a reunion and banquet at the Hotel Statler Wednesday evening, May 25. Of the sixteen members now living, twelve were present. Dr. C. B. Kibler, of Corry, Pa., and Dr. William J. Cronyn, of Milwaukee, were among the banqueters.

### Hospital Appointments.

Members of the class of 1910 have received hospital appointments in accordance with the following schedule:

| | |
|---|---|
| Blum, Raymond J. | St. Mary's, Rochester. |
| Brown, Stanley J. | Auburn City, Auburn. |
| Burke, Thomas J. | Emergency, Buffalo. |
| Culkowski, Anthony S. | German, Buffalo. |
| Duffy, Benedict J. | Sisters of Charity, Buffalo. |
| Eustace, Charles W. | Moses Taylor, Lackawanna. |
| Fakhoury, Elias K. | Prussian, Beirut, Egypt. |
| Goodell, Charles E. | Buffalo General, Buffalo. |
| Greene, Clayton W. | Buffalo General, Buffalo. |
| Grenolds, Walter J. | Erie County, Buffalo. |
| Hartigan, William S. | Erie County, Buffalo. |

Hughes, Ralph R. ............Buffalo General, Buffalo.
Hogan, John, ................Sisters of Charity, Buffalo.
Keyes, Marion A. ............Erie County, Buffalo.
McCall, James M. ............Buffalo General, Buffalo.
McMahon, Michael J. ........St. John's Long Island City.
Nesbitt, Clarence C. ..........Sisters of Charity, Buffalo.
Orvis, Howard A. ...........Buffalo General, Buffalo.
Sperry, Frederick E. .........Sisters of Charity, Buffalo.
Stein, August H. ............Buffalo General, Buffalo.
Stockwell, Raymond W. ......Buffalo General, Buffalo.
Stygall, James H. ............Erie County, Buffalo.
Sullivan, James C. ...........Buffalo General, Buffalo.
Vanderboget, Carlton L. ......General, Seattle, Wash.
Wagner, Albert W. ..........German Deaconess, Buffalo.
Wilkins, Leslie M. ...........Moses Taylor, Lackawanna.

---

THE Carnegie Foundation report on medical education which appeared early in June, has bestirred the faculties of several schools, some of whom have displayed considerable feeling on the subject. The most notable instance is that of one in Saint Louis which proposes to enter suit against the foundation for damages. The report of the council on medical education was made public at the meeting of the American Medical Association at Saint Louis, and is along the same lines as that of the foundation, though less complete and much less drastic. While it is well understood that much improvement is needed to bring the standard of medical education up to a proper level,—a uniform standard,—in this country, yet it is unwise to move so rapidly as to damage well-meaning schools already on the road toward improvement.

---

THE undignified habit of some of the newspaper press of referring to the President as "Bill," or to the former president as "Teddy," in headlines or text, should cease. Even the *Morning Telegraph* (New York), utters a protest against the practice. What sometimes may be said playfully in speech, appears quite out of place in type where it may set a bad example to the young. Let us always, in speech, type or writing, not only treat our Chief Magistrate with respect, but, likewise, all who have held the office.

---

THE Buffalo delegation to the American Medical Association at Saint Louis, assisted by a strong contingent of associates, urgently and cordially invited the association to hold its next meeting in this city. How near they came to success is indicated by the vote in the house of delegates, where all business questions are settled. Buffalo and Los Angeles were placed in nomination, one hundred and nineteen members being present. Of this number Buffalo received fifty-eight votes and Los Angeles sixty-one, thus winning by a majority of three votes. It seems evident that Buffalo will be selected as the place of meeting in 1912, unless some unlucky circumstance occurs in the meantime, or maladroit management causes failure.

---

Calox is a dentifrice that should find its way to the mouth of every person who believes in keeping the teeth free from the germs of decay. The oxygen it contains should make it appeal at once to the physician or dentist, as well as to any man or woman of science or who believes in cleanliness. Calox, indeed, should be found among the toilet belongings of every man or woman of refinement.

---

Dr. F. E. Fronczak, the Health Commissioner, proposes that the antispitting ordinance shall be enforced in Buffalo. It is stated that recently he held a conference with Police Commissioners Zeller and Doherty and Superintendent of Police Regan, in the course of which he informed the police authorities that the ordinance prohibiting spitting on the sidewalks and in public places was not sufficiently inforced and suggested that the police be more active in future in making arrests. It is quite time that something was done to check this filthy and dangerous practice. A few fines would help amazingly.

